# A Human 3D Cardiomyocyte Risk Model to Study the Cardiotoxic Influence of X-rays and Other Noxae in Adults

**DOI:** 10.3390/cells10102608

**Published:** 2021-09-30

**Authors:** Timo Smit, Esther Schickel, Omid Azimzadeh, Christine von Toerne, Oliver Rauh, Sylvia Ritter, Marco Durante, Insa S. Schroeder

**Affiliations:** 1 Biophysics Department, GSI Helmholtzzentrum für Schwerionenforschung GmbH, 64291 Darmstadt, Germany; t.smit@gsi.de (T.S.); e.schickel@gsi.de (E.S.); s.ritter@gsi.de (S.R.); m.durante@gsi.de (M.D.); 2 Biology Department, Technische Universität Darmstadt, 64289 Darmstadt, Germany; rauh@bio.tu-darmstadt.de; 3 Section Radiation Biology, Federal Office for Radiation Protection (BfS), 85764 Neuherberg, Germany; oazimzadeh@bfs.de; 4 Helmholtz Zentrum München-German Research Center for Environmental Health, Institute of Radiation Biology, 85764 Neuherberg, Germany; 5 Helmholtz Zentrum München-German Research Center for Environmental Health, Research Unit Protein Science, 80939 Munich, Germany; vontoerne@helmholtz-muenchen.de; 6 Institute for Condensed Matter Physics, Technische Universität Darmstadt, 64289 Darmstadt, Germany

**Keywords:** cardiomyocytes, maturation, X-rays, stem cell differentiation, risk assessment, structural remodeling, 3D culture

## Abstract

The heart tissue is a potential target of various noxae contributing to the onset of cardiovascular diseases. However, underlying pathophysiological mechanisms are largely unknown. Human stem cell-derived models are promising, but a major concern is cell immaturity when estimating risks for adults. In this study, 3D aggregates of human embryonic stem cell-derived cardiomyocytes were cultivated for 300 days and characterized regarding degree of maturity, structure, and cell composition. Furthermore, effects of ionizing radiation (X-rays, 0.1–2 Gy) on matured aggregates were investigated, representing one of the noxae that are challenging to assess. Video-based functional analyses were correlated to changes in the proteome after irradiation. Cardiomyocytes reached maximum maturity after 100 days in cultivation, judged by α-actinin lengths, and displayed typical multinucleation and branching. At this time, aggregates contained all major cardiac cell types, proven by the patch-clamp technique. Matured and X-ray-irradiated aggregates revealed a subtle increase in beat rates and a more arrhythmic sequence of cellular depolarisation and repolarisation compared to non-irradiated sham controls. The proteome analysis provides first insights into signaling mechanisms contributing to cardiotoxicity. Here, we propose an in vitro model suitable to screen various noxae to target adult cardiotoxicity by preserving all the benefits of a 3D tissue culture.

## 1. Introduction

Cardiovascular disease (CVD) is the number one cause of death worldwide, including a variety of conditions such as ischemic disease, heart attacks, and conduction abnormalities [1]. Various factors such as dietary, metabolic disorders, and genetic predisposition are reported to increase the risk of developing CVD. Environmental factors also contribute to the risk, including radiation [2]. Due to a growing patient population receiving ionizing radiation to treat cancerous diseases, adverse effect concerns have arisen based on clinical observations including conduction system abnormalities, cardiomyopathy and pericarditis [3,4]. Advances in treatment have comprised of a decrease in the received dose and the volume of the exposed heart [5]. However, underlying mechanisms of radiation-induced cardiovascular diseases are still poorly understood and a more precise risk estimation regarding the cardiovascular system is essential for treatment progression. To date, targeting cardiotoxicity of different noxae is challenging due to a lack of reliable and simple assays.

Primary adult human cardiomyocytes (CM) from biopsies are difficult to culture and do not allow high-throughput experiments. Therefore, other model systems are commonly used. The hERG (human ether-a-go-go related gene) in vitro assay, routinely used in preclinical drug testing, detects potential effects on a potassium channel, the inhibition of which leads to arrhythmias. For testing, hERG channels are heterologously expressed in immortalized embryonic kidney cells (HEK 293), which are examined by the patch-clamp technique [6]. Due to the non-cardiac nature of these cells, predictability is questionable and limited only to noxae that inhibit the hERG channel. Furthermore, proarrhythmic effects of drugs are investigated in animal studies, which are difficult to extrapolate to humans due to inter-species differences in ion channel composition, action potential characteristics and biological pathways [6,7]. To overcome this limitation, human pluripotent stem cells (hPSC) are promising as they can differentiate into any cell of the body, including CM [8]. Here, induced pluripotent stem cells (iPSC) derived from various somatic cell sources are prevalently used to generate CM [9,10,11] due to accessibility or patient specific applications. In contrast, well established embryonic stem cell lines (hESC) are utilized in the minority of studies [12,13,14] because of ethical hurdles. Such 2D or 3D stem cell-derived systems are an attractive alternative, but the major concern is their immature phenotype [15,16]. These systems often lack hallmarks of maturation like typical rod shape, multinucleation, as well as sarcomere size and order [17] thereby failing to faithfully recapitulate pathophysiological mechanisms of adults. Such immature cells are widely used in drug discovery pipelines for cardiotoxicity assessment beyond the classical hERG assay. The comprehensive in vitro proarrhythmia assay (CiPA) initiative estimates risks of different chemical compounds by confirming in silico reconstruction of cardiac action potentials and confirms these findings in hPSC-derived CM (hPSC-CM) [10]. The CiPA methods were complemented by the establishment of a cardiac safety index (CSI) estimated by different in vitro readouts regarding contractility and cytotoxicity in immature hPSC-CM [18]. However, these approaches also fail to cover effects of noxae, whose detrimental effects are likely caused beyond chemical induction. More advanced models make use of 3D constructs by assembling a mixture of different CM or co-culture with supporting cells such as fibroblasts and endothelial cells [9] as well as shaped constructs [12] or high-fidelity cardiac organoids in the field of tissue engineering [11]. While these approaches aim to generate more organotypic cultures, the involved complexity hinders the compatibility for standard lab applications. In general, 3D constructs of hPSC-CM have the advantage of maturation and contractility that reflects the main function of the heart compared to 2D culture [19]. Different strategies are being pursued to achieve maturation of hPSC-CM. In addition to mechanical loading and electrical stimulation, long-term culture is promising in terms of morphology, functionality, and electrophysiology [20].

Here, we propose a versatile risk model to study different noxae whose effects are unlikely to be detected by classical cardiotoxicity testing. For this, we used self-organizing 3D clusters of hPSC-CM (hPSC-CMC) derived from embryonic cells, mirroring the main structure and function of the adult myocardium. These clusters were irradiated with X-rays in a range of 0.1–2 Gy at the state of maximum maturation achievable by long-term culture for simple screening of cardiotoxicity using a video-based functional assay. In addition, proteomic assays were performed and gave first insights into underlying pathophysiological mechanisms.

## 2. Materials and Methods

### 2.1. The Cell Lines and Culture Conditions

The human embryonic stem cell line H9-hTnnTz-pGZ-D2 (H9-cTNT) was obtained from WiCell Research Institute (Madison, WI, USA) and was used for all experiments according to the German Stem Cell Act (approval registry number 1710-79-1-4-69, issued by the Robert-Koch-Institute, Zulassungsstelle für Anträge nach dem Stammzellgesetz, Berlin, Germany). This transgenic cell line expresses green fluorescent protein (GFP) under the control of the cardiac troponin-T-promotor to follow cardiac differentiation. Every seven days, cells were passaged on 5 µg/mL human recombinant laminin-521-coated culture dishes (BioLamina, Sundbyberg, Sweden, #LN521) in mTeSR^TM^1 medium (Stemcell Technologies, Vancouver, BC, Canada, #85850) supplemented with 50 µg/mL penicillin and 5 µg/mL streptomycin (Merck, Kenilworth, NJ, USA, #A2212) by using the non-enzymatic ReLeSR^TM^ reagent (Stemcell Technologies, #05872). For this, cells were washed with 2 mL PBS and subsequently rinsed with 200 µL ReLeSR^TM^ per petri dish and incubated for four minutes at 37 °C, detaching primarily pluripotent cells. Detached cells were collected in mTeSR^TM^1 medium and 0.21 × 10^6^ cells were reseeded per laminin coated culture dish (equaling 1 × 10^5^ cells/cm^2^). mTeSR^TM^1 medium was exchanged daily. For experiments, cells in passage 17 to 40 were used.

### 2.2. Differentiation of H9-cTNT into hPSC-CMC

H9cTNT cells were collected during passaging and were seeded on Geltrex^TM^-coated 6-well plates (Life Technologies, #A15696-01); 1.7 × 10^6^ cells per well were plated to reach confluence without spontaneous differentiation after four to seven days in mTeSR^TM^1 medium. Confluent cultures were differentiated according to a modified protocol (Table 1). This protocol alters the WNT signaling pathway to generate cardiac cells based on a protocol introduced by Kadari and colleagues [21]. Here, differentiation into mesoderm was induced by adding 5 µM CHIR99021 (BioVision, Milpitas, CA, USA, #1991-1) for four days, and 25 ng/mL BMB4 (PeproTech, Cranbury, NJ, USA, #120-05) for one day to insulin-free basal medium containing RPMI1640 (Life Technologies, Carlsbad, CA, USA, #61870010) supplemented with insulin-free B-27^TM^ (Life Technologies, #A1895601), 50 µg/mL penicillin and 5 µg/mL streptomycin (Merck, #A2212), and 50 μg/mL l-ascorbic acid (Sigma, St. Louis, MI, USA, #49752). Six to seven days into differentiation, 10 µM XAV939 (Tocris Bioscience, Bristol, UK, #3748) was added to the basal medium to induce cardiac specification. The insulin-free medium was changed to an insulin-containing medium between day seven and 14 post differentiation initiation. Therefore, the medium was supplemented with B-27^TM^ (Life Technologies, #17504044) instead of the insulin-free B-27^TM^ to reduce metabolic stress. Between 14 and 18 days of differentiation, cardiac enrichment medium containing glucose-free RPMI1640 (Life Technologies, #11879020), 50 µg/mL penicillin and 5 µg/mL streptomycin and 4 mM lactate (Sigma, #71718) as energy source, was used to select for CM. After these phases of differentiation and selection, cultures containing beating hPSC-CMC were kept in insulin-free medium to allow further maturation until day 300.

### 2.3. Immunocytochemical Analyses of Cardiac Markers

Individual hPSC-CMC showing spontaneous contraction were detached from the cell culture plates using a scalpel and were fixed in 3.7% formaldehyde solution in PBS^−/−^ for 45 min inside 2 mL reaction tubes. Fixed hPSC-CMC were permeabilized for 30 min with 0.5% TritonX 100 in 1% bovine serum albumin (BSA). Non-specific binding sites were blocked by incubation with 1% BSA for 30 min before incubation with the primary antibody for one hour. The following primary antibodies were used: Anti-α-actinin, sarcomeric (Merck, #A7732); anti-connexin 43/GJA1 (Abcam, Cambridge, UK, #ab11370); anti-ryanodine receptor 2 (Badrilla, Leeds, UK, #a010-31); anti-HCN4 (Abcam, #ab66501); and anti-myosin light chain 2 (Abcam, #ab79935). The secondary antibodies Alexa594 Fluor^TM^ anti-mouse (Merck, #A32744) or anti-rabbit (Merck, #A24343) were also applied for one hour. Between all steps samples were washed in PBS^−/−^. Cell nuclei were stained with 1 µg/mL DAPI for 10 min, rinsed with water and transferred to slides for microscopy using fluorescence mounting medium. For staining the cytoskeleton, fixed hPSC-CMC were incubated with 200 nM Phalloidin-TRITC (R&D Systems, Minneapolis, MN, USA, #915013-10-4) for 30 min. All steps were performed at room temperature. Samples were analyzed on a confocal microscope (Leica DMI 4000 B, Leica Microsystems, Buffalo Grove, IL, USA) using the Leica Application Suite Advanced Fluorescence software (version 2.7.3.9723). For image processing, the open-source software ImageJ for Windows (https://imagej.nih.gov, version 1.50i, accessed on 27 August 2021) was used.

### 2.4. Measurements of α-Actinin Chain Lengths

α-actinin chains (n) from immunocytochemical stainings were measured using the ImageJ program. Per time point >3 hPSC-CMC were analyzed at 50 (n = 64 chain measurements), 100 (n = 85), 200 (n = 106), 250 (n = 67), and 300 (n = 27) days after differentiation initiation. Only chains that were located in their entirety in the focal plane across 10–30 µm spanning Z-stacks were measured.

### 2.5. Patch-Clamp Analysis of Individual Cells

Electrophysiological measurements were performed using the patch-clamp technique in whole-cell configuration. hPSC-CMC were incubated for three to seven hours in 1 mg/mL collagenase type II (StemCell Technologies, #07418) in PBS at 37 °C and 5% CO_2_. Detached cells were seeded on 35 mm plates coated with Geltrex^TM^ (Life Technologies, #A15696-01). For the recording of single cells, patch-pipettes were pulled from Sigmacote^TM^ (Merck, #SL2) coated borosilicate glass capillaries using a single stage glass microelectrode puller (PP-830, Narishige Group, Tokyo, Japan) resulting in pipettes with resistances between 2.5 and 5.5 MΩ. Patch-pipettes were filled with internal solution (in mM: 5 NaCl, 20 KCl, 10 HEPES, 2 MgATP, 125 potassium gluconate, set to pH 7.2 with KOH; 320 mOsmol/kg) and connected via an Ag/AgCl electrode to the head stage of an EPC-9 patch clamp amplifier (HEKA Elektronik GmbH, Lambrecht, Germany). Cells were bathed in external solution (in mM: 140 NaCl, 5.4 KCl, 5 HEPES, 1 MgCl_2_, 1.8 CaCl_2_, 5.5 glucose; set to pH 7.4 with NaOH; 310 mOsmol/kg) and placed on the stage of an inverted microscope. The bath solution was connected via an Ag/AgCl-electrode to the ground. Membrane potentials were recorded in the current-clamp mode, filtered at 10 kHz using a low pass Bessel filter and digitized at a sampling rate of 20 kHz. Data was collected with PatchMaster (HEKA Elektronik GmbH, Lambrecht, Germany) and analyzed with FitMaster (HEKA Elektronik GmbH, Lambrecht, Germany). Liquid junction potentials (LJPs) were calculated using JPCalcWin (School of Medical Sciences, University of New South Wales, Sydney, Australia) and subtracted post recording. Spontaneous action potential-generating cells were measured three times for two minutes in current-clamp for 20 s without current injection at RT. Cells showing no spontaneous action potential were additionally triggered with 2 ms long pulses of the 1.5 fold currents necessary to induce the action potential. Between pulses, cells were clamped to their membrane potential for 500 ms.

### 2.6. X-ray Irradiation of hPSC-CMC

At the time of irradiation, hPSC-CMC were maintained in either 24 well-plates (for ELISA assays and protein sampling) or 96 well plates (for video analyses). X-ray exposure was performed at an Isovolt DS1 X-ray tube (250 kV and 16 mA, 2 Gy/min) at RT. Controls were sham-irradiated and the medium was exchanged immediately after irradiation.

### 2.7. Video-Based Analysis of hPSC-CMC

For video analyses, individual hPSC-CMC were detached from the culture plate using a scalpel and transferred to Geltrex^TM^-coated 96 well plates so that each well only contained a single hPSC-CMC. At the time of transfer, hPSC-CMC were at least 100 days old and maintained in insulin-free medium. To determine the beat rate and rhythmicity of individual hPSC-CMC, the software cBRA (cardiac Beat Rate Analyzer) and the setup presented by Nitsch and colleagues [22] were used. For measurements of external stimuli, the temperature of the microscope-mounted heated plate was adjusted in the range of 32–38 °C (recordings of n = 18 individual hPSC-CMC), or medium spiked with 1 µM each of isoprenaline (Merck, #I6504, n = 15 individual hPSC-CMC), propranolol (Merck, #P0884, n = 15) or verapamil (Merck, #V4629, n = 7) was consecutively added to controls (n = 15) in the order mentioned. Videos were recorded after 10 min of incubation. In between measurements of drugs, hPSC-CMC were washed thoroughly with culture medium. Videos of X-ray irradiated hPSC-CMC were recorded one day before irradiation, and one day, one week (7 days), and one month (28 days) after irradiation. n = 30–50 individual hPSC-CMC were recorded per irradiation dose and time point of n = 3 independent experiments. For the functional evaluation, measurements were taken at 37 °C for one minute per beating hPSC-CMC at a rate of 100 frames per second. Parameters of the video analysis after X-ray irradiation were standardized to the respective measurements before the treatment and standardized to the respective controls for better visuality. Graphs were created with GraphPad Prism for Windows (www.graphpad.com, version 8.0.0, accessed on 27 August 2021) or the open-source software Graph for Windows (http://www.padowan.dk, version 4.4.2, accessed on 27 August 2021). Data were standardized to the respective measurement before irradiation and the respective controls. Statistical analysis was performed using two-sided *t*-test (* *p* ≤ 0.05, ** *p* ≤ 0.01, *** *p* ≤ 0.001).

### 2.8. Interleukin 6 and 8 ELISA Assays

To investigate the pro-inflammatory potential of X-rays on cardiac cells, quarter wells of 6-well culture plates were transplanted to one well of a Geltrex^TM^-coated 24-well plate using a scalpel. At the time of transferring, cells were at least 100 days old. 24-well-plates containing the hPSC-CMC were irradiated with 2 Gy X-rays. Culture medium was incubated for one or three days and collected 1, 3, 6, and 28 days after (sham-) irradiation from n = 6 cultures, pooled and stored at −80 °C until ELISA testing. ELISA assays for the human interleukins IL-6 (Invitrogen, Waltham, MA, USA, #88-7066-88) and IL-8 (Invitrogen, #88-8086-88) were performed with the collected supernatants according to the manufacturer’s instructions. Graphs were generated using GraphPad Prism. Data were normalized to cultivation time of three days in the supernatant and statistical analysis was performed using two-sided *t*-test (* *p* ≤ 0.05, ** *p* ≤ 0.01, *** *p* ≤ 0.001).

### 2.9. Size Measurements

The perimeters of hPSC-CMC, which were covered by the entire image section of the video analysis (n = 23 for 2 Gy X-ray irradiated and n = 22 hPSC-CMC for controls), were measured using the ImageJ program to investigate a hypertrophic effect of X-ray irradiation. Graphs were plotted with GraphPad Prism for Windows.

### 2.10. Proteomic Analysis

To investigate the effects of X-rays on the hPSC-CMC proteome, the cultures were transferred by scalpel from the donor plate into 24 well-plates and irradiated with 2 Gy of X-rays. At this time, the hPSC-CMC were 60 days old. Samples were analyzed by label free quantitative proteomics. For this purpose, five biological replicates each were collected one day before (d-1), one day (d1), and one week (d7) after irradiation. Cells were lysed by RIPA (Thermo Fischer, #89900) containing PhosSTOP^TM^ (Merck, #906845001) and cOmplete^TM^ (Merck, #4693159001). After shaking for 30 min at 4 °C, the cells were sonicated in ice water for 30 s and incubated again for 15 min at 4 °C while shaking. After centrifugation at 20,000× *g* at 4 °C, the supernatant was collected and stored at −20° for further experiments.

10 µg of protein lysate from each replicate were enzymatically digested using a modified filter-aided sample preparation (FASP) protocol as described by [23,24]. Peptides were stored at −20 °C until mass spectrometry (MS) measurement. MS measurement was performed in data-dependent (DDA) mode. MS data were acquired on a Q Exactive (QE) high field (HF) mass spectrometer (Thermo Fisher Scientific Inc., Waltham, MA, USA) as described before [25]. Proteome Discoverer 2.4 software (Thermo Fisher Scientific; version 2.4.1.15) was used for peptide and protein identification via a database search (Sequest HT search engine) against Swissprot mouse data base (Release 2020_02, 17061 sequences), considering full tryptic specificity, allowing for up to one missed tryptic cleavage sites, precursor mass tolerance 10 ppm, fragment mass tolerance 0.02 Da. Carbamidomethylation of Cys was set as a static modification. Dynamic modifications included deamidation of Asn and Gln, oxidation of Met; and a combination of Met loss with acetylation on protein N-terminus. Percolator was used for validating peptide spectrum matches and peptides, accepting only the top-scoring hit for each spectrum, and satisfying the cutoff values for FDR < 1%, and posterior error probability < 0.01. The final list of proteins complied with the strict parsimony principle.

The quantification of proteins was based on the area value of the abundance values for unique plus razor peptides. Abundance values were normalized in a retention time-dependent manner to account for sample loading errors. The protein abundances were calculated summing up the abundance values for admissible peptides. The final protein ratio was calculated using median abundance values of five replicate analyses each. The statistical significance of the ratio change was ascertained employing the T-test approach described in [26] which is based on the presumption that we look for expression changes for proteins that are just a few in comparison to the number of total proteins being quantified. The quantification variability of the non-changing “background” proteins can be used to infer, which proteins change their expression in a statistically significant manner. Proteins identified with at least two unique peptides and with fold change ratios greater than 1.50-fold or less than 0.66-fold (adjusted *p* < 0.05) were defined as being significantly differentially expressed. The analyses of protein–protein interaction and signaling networks were performed by the analysis tool g:Profiler (version e104_eg51_p15_3922dba) [27]. Graphs were created with the GraphPad Prism program.

## 3. Results

The present study aimed to investigate the suitability of aged hPSC-CMC for the risk assessment of different noxae through an in-depth characterization of key features mirroring a physiological and mature myocardium in vitro. Utilizing X-ray radiation as one of those noxae, whose effects are challenging to assess in human adults, our investigations, which included assays regarding functionality, pro-inflammatory responses, and proteome changes, demonstrate the applicability of hPSC-CMC for cardiotoxicity studies.

### 3.1. Prolonged Cultivation Leads to Maturation of CM within hPSC-CMC

**Figure 1:**
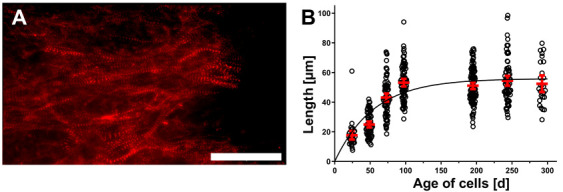
hPSC derived CM mature within the self-organizing 3D network by extended cultivation time. (**A**) Typical striated pattern of immunohistochemically stained α-actinin (ACTN) after 75 days of cultivation. Scale bar: 50 µm. (**B**) Measurements of α-actinin chain lengths in µm over a cultivation period of 300 days after differentiation initiation. Circles show multiple individual measurements (n = 27–106) of >3 hPSC-CMC per time point. Line indicates nonlinear fit of data. Red marks represent the means ± 95% CI.

**Figure 2:**
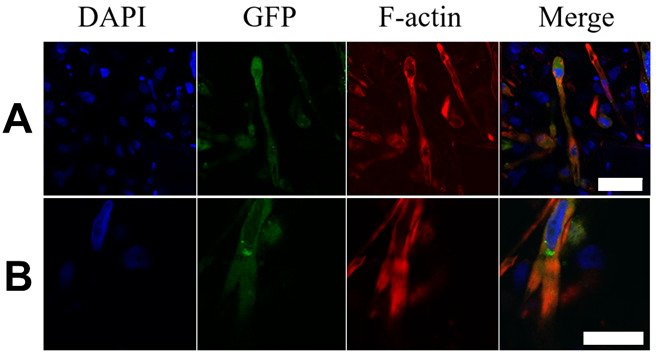
hPSC-CM show distinct characteristics of maturation after 100 days in culture. The staining of 100-day-old hPSC-CMC shows the DAPI signal of the cell nuclei (blue), cTNT-GFP CM control (GFP, green), F-actin (Phalloidin-TRITC, red), and a merge of these channels. Exemplary CM with (**A**) multiple nuclei and (**B**) typical branching are shown. Scale bars: 50 µm (**A**), 25 µm (**B**).

To estimate the degree of maturation of the hPSC-CM, the Z-disc protein α-actinin was stained at different time points (25–100 days) after the start of differentiation. Staining revealed the typical pattern of α-actinin chains within the hPSC-CMC (Figure 1A). With increasing time in culture, the organization of the Z-disk structures became more ordered, so that after 75 days anisotropic structures were present. α-actinin chain lengths increased from a mean of 17.8 µm at day 25 to a mean of 54.4 µm after 100 days and remained at this level for the remainder of the 300-day observation period (Figure 1B). In addition, individual CM possessed other hallmarks of maturation at that time including multinucleation and the typical branching of adult cells, supporting the observation of a maturation process (Figure 2).

### 3.2. Matured hPSC-CMC Exhibit Key-Markers of the Heart and Mirror the Myocardial Structure

**Figure 3:**
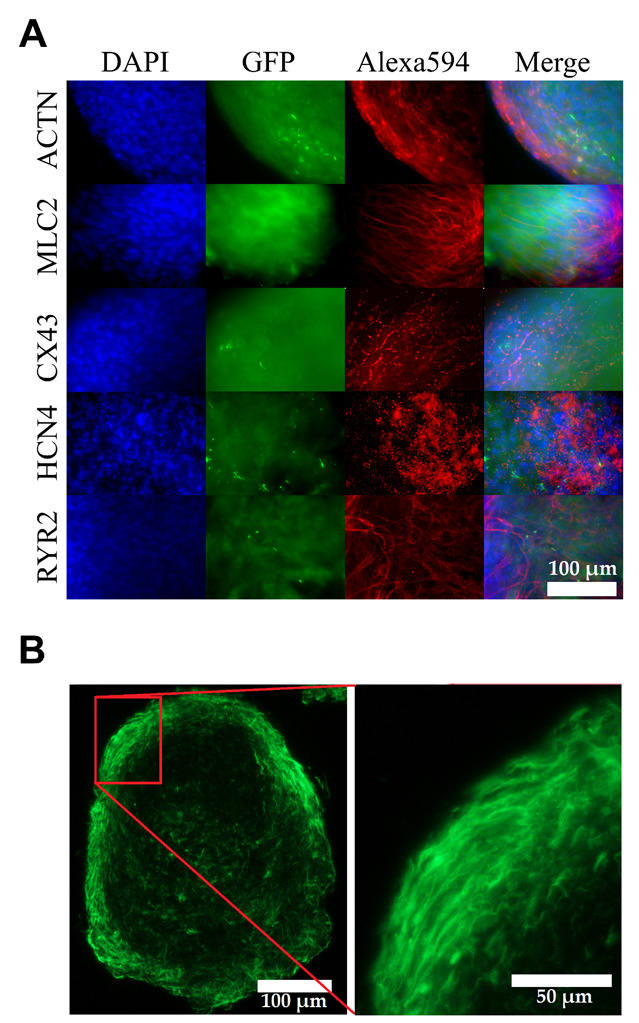
hPSC-CMC express the most important cardiac proteins after 100 days of differentiation and show discrete cell alignment. (**A**) Immunohistochemical staining of whole hPSC-CMC. The proteins ACTN, MLC2; CX43, HCN4, and RYR2 (vertical) are shown in red. Cell nuclei were counterstained with DAPI (blue), GFP (green) was expressed intrinsically under the control of the cTNT promoter; the outer left column shows a merge of these channels. (**B**) Unstained cross-section shows the green cTNT-GFP signal.

To further characterize the hPSC-CMC, important cardiac specific proteins were stained by immunohistochemistry at 100 days after differentiation initiation. Besides α-actinin (ACTN), Myosin light chain 2 (MLC2) was detected as part of the contractile apparatus (Figure 3A). Furthermore, the hPSC-CMC expressed other markers essential for the function and structure of a proper model system, including ryanodine receptor 2 (RYR2), which regulates intracellular calcium release and is responsible for excitation–contraction coupling. Likewise, potassium/sodium hyperpolarization-activated cyclic nucleotide-gated channel 4 (HCN4), which induces the spontaneous generation of action potentials and thus the automaticity of contracting, as well as connexin 43 (CX43), which electrically interconnects the individual cells and thereby builds a functional syncytium for the transmission of depolarization, were found. cTNT-GFP staining revealed an ordered and anisotropic cell alignment following the spherical shape of the hPSC-CMC, mimicking the myocardial structure (Figure 3B). In addition to contraction, this structure is critically involved in the directed transmission of depolarization.

### 3.3. Matured hPSC-CMC Contain All Major Cardiac Cell Types Featuring a Physiologic Cell Composition

**Figure 4:**
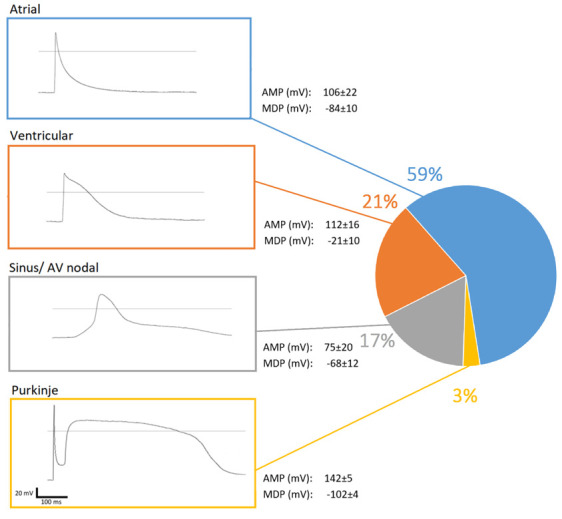
hPSC-CMC are composed of all major CM types. Examples of recordings using the patch-clamp technique in the whole-cell configuration (respective boxes) show the characteristic action potential kinetics by which the different CM types were assigned. Assigned voltages of the amplitude of the action potential (AMP) and minimum diastolic membrane potential (MDP) are the mean values of all measurements of the respective cell type. Percentages of characteristic cardiac cell types are shown in the pie chart (n = 33). AV = atrioventricular.

In order to investigate the cell composition within these hPSC-CMC, action potentials of individual cells were recorded using the patch-clamp technique in the whole-cell configuration after mild dissolving of the hPSC-CMC using a collagenase solution. Cells within the hPSC-CMC exhibited either spontaneous or current-injection triggered action potentials. The specific and characteristic action potential kinetics of 33 individual cells were allocated to the respective cardiac cell types. Thereby all major cell types were identified (Figure 4). Atrial cells were the most common (59%), followed by ventricular cells (21%) and cells of the sinus and atrioventricular nodes (17%) as well as Purkinje cells (3%).

### 3.4. External Stimuli Alter the Contraction of hPSC-CMC in a Physiologic Manner

**Figure 5:**
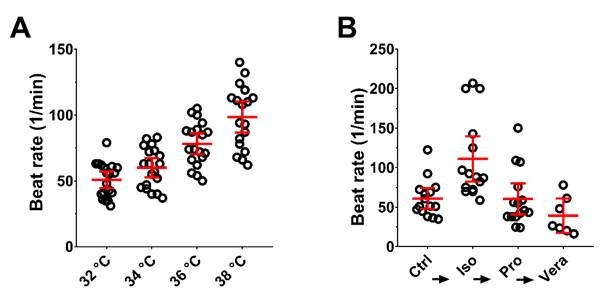
hPSC-CMC respond with altered beat rate to external stimuli. (**A**) Beat rate of single hPSC-CMC (n = 18) in the temperature range of 32–38 °C. (**B**) Beat rate of hPSC-CMC after subsequent external adding of 1 µm of the drugs isoproterenol (Iso, n = 15), propranolol (Pro, n = 15), and verapamil (Vera, n = 7) to untreated controls (Ctrl, n = 15). Circles show individual measurements; red marks show the mean ± 95% CI.

hPSC-CMC responded physiologically to different external stimuli, such as a gradual increase in the temperature from 32 to 38 °C, which was followed by an acceleration of the beat rate from about 50 to 100 beats per minute (Figure 5A). Adding the cardiac pharmaceutical isoproterenol, a beta-adrenergic agonist homologous to adrenalin, doubled the beat rate from about 60 to 120 beats per minute (Figure 5B). The corresponding antagonist propranolol was added subsequently and lowered the beat rate back to the control level (about 60 beats per minute). After addition of verapamil, a calcium antagonist, it decreased below the control level to about 35 beats per minute.

### 3.5. X-ray Irradiation Induces Subtle Pacing and Arrhythmic Manifestations in Matured hPSC-CMC

**Figure 6:**
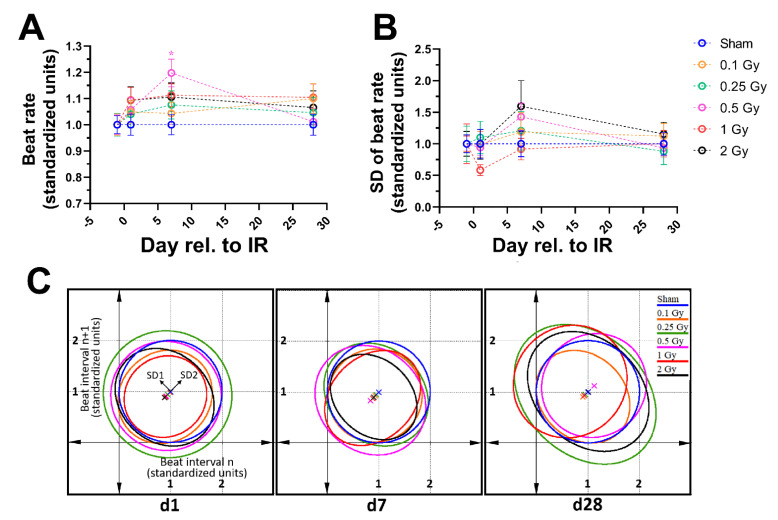
Functional analysis of beating hPSC-CMC reveals subtle changes in beat frequency and rhythmicity. (**A**) Beat rate and (**B**) standard deviation of individual beat rates. One-minute videos of beating hPSC-CMC were taken 1 day prior, and 1, 7, and 28 days post irradiation with different doses (0.1; 0.25; 0.5; 1; 2 Gy) of X-rays. Data were standardized to the respective measurements before irradiation and the respective controls and are shown as mean ± SEM. * *p* ≤ 0.05 using two-sided *t*-test. (**C**) Poincare plots of the measured groups 1 (d1), 7 (d7), and 28 days (d28) after irradiation. Beat intervals were normalized to the respective controls and plotted as a function of the preceding interval (N = 30–50 individual measurements of n = 3 independent experiments per irradiation dose and time point).

To investigate the effects of X-rays on the function of matured hPSC-CMC, these were irradiated with various doses ranging from 0.1 to 2 Gy X-rays and video-based analyses were performed. In the observation course of one month, in all irradiated groups the beat rates increased slightly compared to the controls, although this effect was very small and only significant for 0.5 Gy after seven days (Figure 6A). Even in the group exposed to the lowest dose (0.1 Gy X-rays), hPSC-CMC displayed this slightly increased pace over the entire observation period of one month. While subtle, the changes indicate disturbances in mechanisms controlling the beat frequency. Seven days after irradiation, the standard deviations of individual beat rates increased mostly dose-dependent, indicating a poorer beat regularity compared to the control (Figure 6B). However, 28 days after irradiation the rhythmicity recovered to levels seen in the control. Poincare plots revealed that the heart rate variability one day and seven days after the irradiation comprised equally of SD1 (short-term variability) and SD2 (long-term variability). However, after 28 days, a pronounced preference of SD1 was apparent, indicating short-term arrhythmic manifestations (Figure 6C).

### 3.6. A Moderate Dose of 2 Gy X-rays Induces Neither a Interleukin Storm nor Hypertrophic Growth in Matured hPSC-CMC

**Figure 7:**
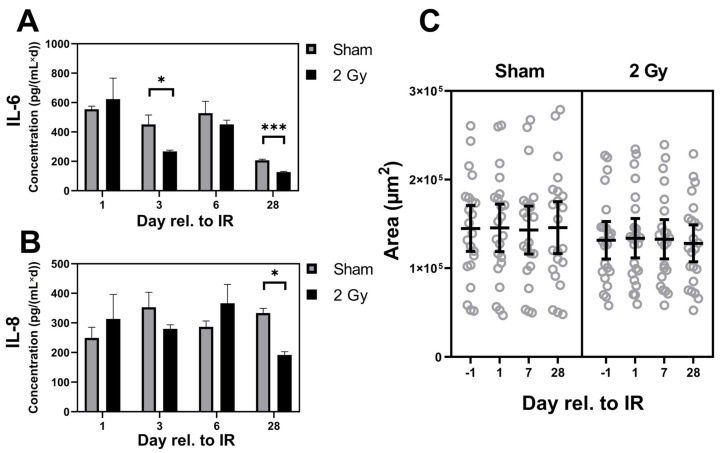
X-ray irradiation (2 Gy) does not trigger excess interleukin secretion or hypertrophic morphology in hPSC-CMC. (**A**,**B**) Secretion of IL-6 and IL-8 1, 3, 6, and 28 days after exposure of hPSC-CMC cultures (n = 6, pooled for analysis) to 2 Gy of X-rays. Data were normalized to a cultivation time of 3 days in the supernatant and shown as mean ± SD. * *p* ≤ 0.05, *** *p* ≤ 0.001 using two-sided *t*-test. (**C**) Area of individual hPSC-CMC (circles, n = 22–23) 1 day prior and 1, 7, and 28 days post irradiation with 2 Gy of X-rays. Data shows the mean ± 95% CI.

As a next step, the inflammatory potential after irradiation with a moderate dose of 2 Gy X-rays was investigated, as it was reported that high radiation doses induce inflammation of the heart [28], represented by an interleukin storm. Supernatants of six biologically replicate samples were pooled and ELISA assays for the interleukins IL-6 and IL-8 were performed 1, 3, 6, and 28 days after irradiation. No excess secretion pattern could be detected within the observation period. On the contrary, compared to the control, a significant decrease of interleukin concentrations was measured after three and six days (IL-6) and 28 days (IL-8), respectively (Figure 7A). Interleukins play a central role in the pathophysiology of cardiovascular diseases, one of them being hypertrophy leading to a swelling of the heart’s muscle. As they were still detectable even in irradiated samples, the size of hPSC-CMC was measured up to one month after 2 Gy X-rays to investigate a potential hypertrophic effect of the radiation. However, no swelling was detected compared to the respective controls during the entire observation period (Figure 7B).

### 3.7. Proteome Changes after X-ray Irradiation Pointing towards a Fast Structural Remodeling and Disease-Related Signaling

**Figure 8:**
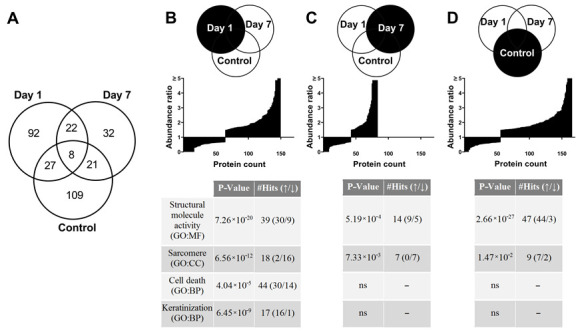
Moderate 2 Gy X-ray irradiation induces a rapid change in the proteome of hPSC-CMC. (**A**) Venn diagram showing the distribution of significantly deregulated (1.5 fold; adj. *p* value ≤ 0.05) proteins 1 day or 7 days after 2 Gy X-rays compared to the respective control. (**B**–**D**) Comparison of the three complete proteome profiles tested. Day 1: 1-day post-irradiation proteome tested against the respective control; Day 7: 7-day post-irradiation proteome tested against the respective control; Control: 7-day control proteome tested against the 1-day control. ns: Not significant.

**Figure 9:**
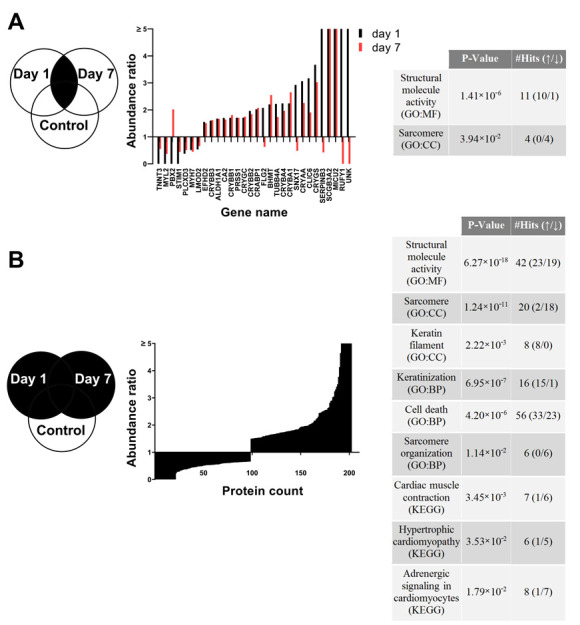
Robust processes of the proteome change induced by 2 Gy X-ray irradiation in hPSC-CMC. (**A**) Abundance ratios of the 30 shared and significantly deregulated (1.5 fold; adj. *p* value ≤ 0.05) proteins 1 day and 7 days after irradiation and associated functional analysis. (**B**) The abundance ratios of all 202 proteins significantly deregulated 1 day and 7 days after irradiation and associated functional analysis.

To identify changes in signaling pathways contributing to the cell response to irradiation, label-free proteomics analyses were performed one day and seven days after irradiation with 2 Gy X-rays. Irradiated samples were tested against controls of the same time point. In addition, controls of day seven were tested against controls of day one to monitor cell growth over the week of observation. In total, 4853 proteins were identified, whereby the effect of 2 Gy X-rays was stronger on proteome changes one day (149 significantly differentially expressed proteins) than seven days (83 proteins) after irradiation (Figure 8A). Comparison of the three profiles tested showed that proteins involved in structural molecular activities were predominantly up-regulated in all groups using enrichment analysis. Here, corresponding proteins changed more in the control (*p* = 2.663 × 10^−27^) and one day after irradiation (*p* = 7.258 × 10^−20^) compared to seven days post irradiation (*p* = 5.188 × 10^−4^). Furthermore, sarcomere proteins were up-regulated in the control during the week of observation (*p* = 1.471 × 10^−2^). In contrast, after irradiation, these were significantly down-regulated, with a stronger effect one day after irradiation (*p* = 6.562 × 10^−12^) than after one week (*p* = 7.333 × 10^−3^). Proteins involved in cell death were detected as significantly deregulated (*p* = 4.044 × 10^−5^) one day after irradiation, but not after seven days or in the control. In addition, proteins involved in keratinization (*p* = 6.446 × 10^−9^) followed the same pattern (Figure 8B–D). To identify persistent changes induced by X-rays, proteins equally deregulated one day and seven days after irradiation were analyzed. Of these 30 significantly deregulated proteins, 11 are involved in structural molecule activity (*p* = 1.413 × 10^−6^) and were strongly up-regulated, including proteins of the crystallin family (CRYBB1, CRYGC, CRYBB2, CRYBA4, CRYBA1, CRYAA, CRYGS). At both time points, proteins of the contractile sarcomere (TNNT3, MYL2, MYH7, LMOD2) were down-regulated (Figure 9A). Loss of contractile units is further supported by analysis of all significantly deregulated proteins at both time points to capture joint processes of the changed proteomes over the week post irradiation (Figure 9B). In this analysis, 202 significantly deregulated proteins were identified with almost equal distribution of up-regulation (104 proteins) and down-regulation (98 proteins). Here, 18 of the 20 sarcomeric proteins found (*p* = 1.24 × 10^−11^) were down-regulated beside differentially expressed proteins with structural molecule activity (*p* = 6.266 × 10^−18^). Further enrichment analysis revealed the biological process of sarcomere organization and proteins involved in cell death signaling (*p* = 4.203 × 10^−6^), as well as a cluster of keratins involved in the process of keratinization (*p* = 6.954 × 10^−7^), including keratin filaments (KRT1, KRT2, KRT5, KRT6A, KERT6B, KRT14, KRT77; KRT 78). KEGG signaling pathway analysis presents cardiac muscle contraction (*p* = 3.453 × 10^−3^) and disease related changes involved in hypertrophic cardiomyopathy (*p* = 3.533 × 10^−2^). Eight mainly down-regulated proteins are involved in the adrenergic signaling pathway in CM (*p* = 1.794 × 10^−2^), which mainly regulates the frequency of beating (Figure 9B).

## 4. Discussion

Human stem cell-derived 3D models of different tissues are attractive to use for risk assessment by external manipulation of different noxae, but are not yet used to their full potential in in vitro toxicity testing [29]. The generation of CM from pluripotent cells has been successfully established by several laboratories, but some aspects still need to be considered for their meaningful application. CM derived from iPSC, which can be generated from various somatic cell sources applying a plethora of reprogramming methods, are an attractive tool to study genetically induced cardiomyopathies and patient-specific reactions to cardiotoxic agents. However, in contrast to hESC, iPSC can acquire genetic aberrations and altered epigenetic profiles during or after the reprogramming process (reviewed in [30]) that may render CM derived from these sources unsuitable for toxicity testing. For example, iPSC-CM can re-express transgenes such as NANOG and OCT4 as an artefact of the reprogramming process [31]. This can lead to dedifferentiation and is a risk in long-term culture. Furthermore, CM from embryonic and somatic cells differ in physiology, with hESC-CM beating faster [31] and the handling of Ca^2+^ being closer to the adult phenotype compared to iPSC-CM [32]. Thus, the use of hESC-CM as carried out in this study may improve the reliability of the generated data. However, both hESC-CM and iPSC-CM in general display a rather immature phenotype [8,33] and their predictability for the adult myocardium is limited. Therefore, we performed long-term cultivation to achieve a cell maturation process during a 300-day long period after the start of differentiation. During the 300-day long observation, we detected an increase in the cell spanning α -actinin chain lengths until 100 days of cultivation with a mean of 55.9 µm and anisotropic patterning. This is a strong evidence for a maturation process as is accompanied by cell growth and order of the cells-spanning sarcomeric structures. Thus, the length of 10 to 30 µm for matured (>35 days) hPSC-CM described in the literature was exceeded by the CM presented in this work after 100 days in 3D culture, approaching the range of adult CM, which are typically between 10 and 150 µm [34]. Lundy et al. [35] cultivated hPSC-CM for up to 120 days by reseeding enzymatically dissolved CM cultures on glass coverslips observing single cells. In the course of 120 days the cells elongated, myofibrillar alignment was more anisotropic and the fraction of multinucleated cells increased by 10-fold testing early (20–40 days) versus late-stage CM (80–120 days). Our data shows that all these morphological maturation processes take place in the hPSC-CMC culture within 100 days. In addition, we found the typical interconnecting branching of CM to be present in the 3D culture. However, no further elongation of cells was found after 100 days, suggesting that the maximum maturity is achieved at that time.

Our electrophysiological measurements revealed mean AMPs (MDPs) of 112, 106, and 75 mV for classified ventricular, atrial, and sinus-/atrioventricular nodal cells. This is in accordance to the properties of adult mammalian cells with 110–120 mV for ventricular and atrial cells and 60–80 mV for sinus-/atrioventricular nodal cells, respectively [36]. Our morphological and electrophysiology data suggest that hPSC-CM are capable of an intrinsic maturation process, carried out by time-depending signaling pathways. The fact that the action potential kinetics revealed all major CM types present within hPSC-CMC underlines the superiority of a 3D culture. Here, the electro/chemical signals are transmitted and processed by different CM types in a more complex physiological context compared to 2D cultures. This makes the functional analyses as a key feature of this model more trustworthy.

To measure the clinical relevant functionality of the contraction, optical techniques often rely on fluorescent protein dyes for calcium or voltage sensing, but loading target cells with dye might alter the electrophysiological properties and potentially bias results [37]. We made use of dye-free video analyses, presented in [22]. By doing so, we could demonstrate that radiation induces subtle changes in CM function by increasing the beat rate in a mostly dose-dependent manner and decreasing rhythmicity. Similar observations of deregulated beat rates have been reported in the literature. Pacing of primary chicken CM was observed after X-ray exposure, where doses below 1 Gy showed a smaller increase in beat frequency than higher doses up to 7 Gy [38]. Contrary, CM derived from induced hPSC showed a decrease in beat rates two days after 5 to 10 Gy X-rays [39]. These controversial effects are probably the results of inter-species differences. However, comparing the hPSC data with our observation suggests that there is a decrease in the beat frequency at higher doses and an increase at low doses, indicating that X-ray effects may not be comparable between dose ranges. As the direction of change is completely opposing, potentially unrelated or contrary pathways might be triggered.

First insights using proteome data after moderate 2 Gy of X-rays in our studies point towards an at least partial impingement of the adrenergic signaling pathway cascade, consequently resulting in a dysregulation of the beat rate. Beyond that, disruption in electrical homeostasis caused by alterations of ion channel kinetics would ultimately result in detrimental functional changes. Any modification (i.e., of the delayed-rectifier K^+^ or L-type Ca^2+^ current) changes electrophysiological properties in an arrhythmogenic manner [40]. Such electrical disruption cannot be ruled out since irradiation with 1 Gy X-rays is reported to disturb intercellular ion homeostasis by elevating cytosolic Ca^2+^ and further activate K^+^ channels in human HEK293 and A549 cells [41], potentially contributing to the observed changes in function.

Nevertheless, the summary of our protein data draws a picture of structural remodeling in which contractive proteins become less abundant and proteins with a structural molecular function are broadly deregulated. Similar cellular responses were observed in total body irradiated mice, where 3 Gy gamma radiation led to a remodeling of cardiac tissue concomitant with degradation of proteins involved in contraction and cytoskeletal reorganization [42]. These changes probably are a result of oxidative stress response induced by radiation [42]. Expressed members of the aB-crystallin family after irradiation support the assumption of a rearrangement process. These proteins are known to play a role in cytoskeletal remodeling during cardiac development and in differentiated tissues by acting as small heat shock proteins stabilizing various filaments and being present in normal and stress conditions [43]. The keratins found after irradiation in the present study may serve as intermediate filaments in a physiological context. These are expressed during cardiogenesis and may have a cardioprotective effect when cardiac desmin filaments are not sufficiently present [44]. In this context, a keratin network could serve as a countermeasure to the loss of sarcomere structures after X-ray exposure. In addition, proteome changes in the presented work associated with the disease of hypertrophic cardiomyopathy were found.

However, after 2 Gy of X-rays we could not detect disease-related excess secretion of pro-inflammatory cytokines in hPSC-CMC, which ultimately lead to hypertrophy [45]. Radiotherapy to the chest in contrast induces chronic excess secretion of interleukins IL-6 and IL-8, potentially manifesting adverse late effects in the heart [46]. These interleukins are predominantly released by endothelial cells as mediators of subsequent inflammatory processes [47], but also CM are involved in pro-inflammatory signaling. IL-6 and IL-8 are apparent in the myocardium mediating acute inflammation formation [28] and hPSC-CM in vitro responded to ischemic conditions with increased levels of IL-6 and IL-8 similar to observations in vivo [28]. In contrast, we measured a decrease in IL-6 and IL-8 release after exposure of hPSC-CMC to 2 Gy X-rays. Sequela of which are unpredictable due to the pleiotropic nature of these cytokines being involved in a variety of biological effects. Consequently, we did not observe hypertrophic morphology of the hPSC-CMC after 2 Gy X-ray irradiation, although the proteomic data pointed towards cardiomyopathy signaling. Therefore, it is to be assumed that mainly pre-symptomatic effects of radiation are detected with the presented model. The preceding asymptomatic changes may evolve into disease later in life, supported by epidemiological data showing that the heart responds very late to radiation even in the very low dose range [48,49].

Little is known about mechanisms leading to a late onset of cardiovascular disease. For the heart as an organ at risk, the impact of different radiation qualities that patients undergoing particle radiotherapy face [50] or that are considered during manned spaceflights [51] has yet to be accurately estimated. Only then can these fields progress and an effective treatment to counteract late radiation-induced heart disease can be developed. The need to link signaling pathway analyses to functional responses is to be highlighted in this respect.

Certainly, the predictive power of the presented model is limited to CM by mirroring the key components of the mature myocardium, but not the whole organ. To cover a broader spectrum of cardiac reactions to noxae, the crosstalk between CM and other cell types such as fibroblasts and endothelial cells needs to be addressed. This is achievable by the co-culture of CM with these cell types. For instance, Beachamp et al. reported on the successful 3D co-culture of cardiac fibroblasts with human iPSC-CM [52]. The authors observed among other things a faster spontaneous contraction rate, but no arrhythmogenic effects caused by the fibroblasts. Our model can thus be extended and tailored to a particular research scope.

In conclusion, we have generated an in vitro tool with good transferability to human adults. By prolonging the cultivation time, little technical effort is involved to sufficiently mature hPSC-CMC for cardiotoxicity studies. We pinpointed this process to be limited to about 100 days in culture. Further elaborate protocols reviewed in [20] may be required to move beyond the presented maturation state including mechanical loading and electrical stimulation. Furthermore, we demonstrated that comprehensive studies of the function by video and secretion analysis, as well as measuring proteomic changes in hPSC-CMC, can extend classical toxicity testing. Thereby, potential cardiac risks of various noxae can be estimated adequately in a physiological self-organizing, but not overly complex, engineered system.

## Figures and Tables

**Table 1:** Media composition and layout of the differentiation protocol to generate hPSC-CMC, according to [21] with modifications.

Day	Basal Medium	Supplements
0	RPMI1640 B27 without insulin 50 μg/mL l-ascorbic acid 50 µg/mL penicillin and 5 µg/mL streptomycin	CHIR99021 BMP4
1	RPMI1640 B27 without insulin 50 μg/mL l-ascorbic acid 50 µg/mL penicillin and 5 µg/mL streptomycin	CHIR99021
2	RPMI1640 B27 without insulin 50 μg/mL l-ascorbic acid 50 µg/mL penicillin and 5 µg/mL streptomycin	-
3–6	RPMI1640 B27 without insulin 50 μg/mL l-ascorbic acid 50 µg/mL penicillin and 5 µg/mL streptomycin	10 μM XAV939
7	RPMI1640 B27 without insulin 50 μg/mL l-ascorbic acid 50 µg/mL penicillin and 5 µg/mL streptomycin	-
8–13	RPMI 1640 without glucose 50 µg/mL penicillin and 5 µg/mL streptomycin	4 mM sodium l-lactate
14–18	RPMI1640 B27 with insulin 50 μg/mL l-ascorbic acid 50 µg/mL penicillin and 5 µg/mL streptomycin	-
19–300	RPMI1640 B27 without insulin 50 μg/mL l-ascorbic acid 50 µg/mL penicillin and 5 µg/mL streptomycin	-

## Data Availability

The mass spectrometry proteomics data have been deposited to the ProteomeXchange Consortium via the PRIDE [53] partner repository with the dataset identifier PXD028099.
